# Balancing high accrual and ethical recruitment in paediatric oncology: a qualitative study of the 'look and feel' of clinical trial discussions

**DOI:** 10.1186/1471-2288-10-101

**Published:** 2010-10-22

**Authors:** Lucie MT Byrne-Davis, Peter Salmon, Katja Gravenhorst, Tim OB Eden, Bridget Young

**Affiliations:** 1 University of Manchester, Education & Research Centre, University Hospital of South Manchester, Southmoor Road, Manchester, M23 9LT UK; 2 Institute of Psychology, Health and Society, University of Liverpool, Whelan Building, Brownlow Hill, Liverpool, UK; 3 Academic Unit of Paediatric and Adolescent Oncology, School of Cancer Studies and Enabling Sciences, University of Manchester, c/o Christie Hospital, Manchester, UK

## Abstract

**Background:**

High accrual to clinical trials enables new treatment strategies to be tested rapidly, accurately and with generalisability. Ethical standards also must be high so that participation is voluntary and informed. However, this can be difficult to achieve in trials with complex designs and in those which are closely embedded in clinical practice. Optimal recruitment requires a balance of both ethical and accrual considerations. In the context of a trial of stratified treatments for children with acute lymphoblastic leukaemia (UKALL2003) we examined how recruitment looked to an observer and how it felt to the parents, to identify how doctors' communication could promote or inhibit optimal recruitment.

**Methods:**

We audio-recorded, transcribed and analysed routine doctor-patient consultations (n = 20) and interviews between researchers and parents (n = 30 parents) across six UK treatment centres. Analysis was informed by the constant comparative method. For consultation transcripts, analysis focussed on how doctors presented the trial. We compared this with analysis of the interview transcripts which focussed on parents' perceptions and understanding of the trial.

**Results:**

Parents and doctors discussed the trial in most consultations, even those that did not involve a decision about randomisation. Doctors used language allying them both with the trial and with the parent, indicating that they were both an 'investigator' and a 'clinician'. They presented the trial both as an empirical study with a scientific imperative and also as offering personalisation of treatment for the child. Parents appeared to understand that trial involvement was voluntary, that it was different from routine care and that they could withdraw from the trial at any time. Some were confused about the significance of the MRD test and the personalisation of treatment.

**Conclusions:**

Doctors communicated in ways that generally promoted optimal recruitment, indicating that trials can be embedded into clinical practice. However, parents were unclear about some details of the trial's rationale, suggesting that recruitment to trials with complicated designs, such as those involving stratified treatments, might need enhanced explanation.

## Background

Clinical trials are the gold standard by which new treatments can be reliably tested against current therapies in an attempt to optimise treatment. Paediatric oncology has a tradition of high accrual to trials, although there is considerable variation between trials of treatments for different types of cancer [1-3]. High accrual to clinical trials in childhood cancer is crucial if improvements in treatment are to be maintained, but it is important also to ensure recruitment is conducted in a way that is appropriate for both families and practitioners and is ethically sound. High recruitment is not, therefore, the ultimate goal, but rather it is "optimal recruitment", which takes into account that it is essential that families are able to make informed decisions and to decide themselves whether to enter their child into a trial or not [4].

Certain elements of the way that recruitment is conducted in such trials have been proposed to promote high accrual [3]. These include: that the research question is one that physicians and families consider important and that both parties are comfortable with the clinical and personal equipoise of the study (i.e., that they believe that there is no clear evidence that one treatment is better than another *per se *or for the individual child). Communication between the doctor and decision maker (typically a parent) about the trial has also been shown to be important [5], ensuring, for example, that information about the trial is personal, tailored and timely. It has been proposed that the focus of the consultation in which a trial is discussed should be coherent to parents [3,6]. If a parent believes that a consultation's purpose is to discuss specific results or treatment they may perceive the discussion of a trial as an unwelcome deviation. Throughout recruitment, parents should feel that the doctor gives priority to their child's care over the scientific imperative of the trial and that if trial continuation brought significant physical or emotional cost, the doctor would withdraw the child.

It has been proposed that, before parents give consent for their child to participate, they should be aware: 1) that the trial is different from routine clinical practice, 2) that the treatment is randomly allocated due to uncertainty about which treatment is best, and 3) of the risks, benefits and the right to withdraw from the trial [3,7].

Trial recruitment processes vary between specialities and trials and each situation brings its own specific procedural and ethical challenges. Particular challenges arise in paediatrics linked to children's vulnerability, and legal frameworks that require parents, not patients, to provide consent for entry to a trial [8]. Concerns have been voiced about doctors recruiting patients for whom they have direct clinical responsibility due to a conflict between being both an investigator and the patient's clinician [9,10]. In paediatric oncology it is often the physician with clinical responsibility for the child who approaches the parents about participating in a trial and there is a strong interwoven relationship between routine clinical care and clinical trials. This has been proposed as advantageous for accrual [11] but also as an ethical challenge [9], with evidence in paediatric oncology that parents can struggle to distinguish between treatment that is standard practice and treatment that is part of a trial [12,13]. It has, therefore, been suggested that, in the context of a dependent family-practitioner relationship, high rates of accrual are gained at the expense of informed consent and voluntariness [14].

We investigated how recruitment was conducted in the context of a clinical trial of treatment for childhood leukaemia by focussing on how physicians negotiated the tensions between ensuring high accrual and maintaining high ethical standards. The trial had a complex design with two randomisation points and was investigating treatment stratification. We examined the conduct of recruitment from two perspectives: how recruitment 'looked' to the observer and also how it 'felt' to the parent. By examining these two perspectives simultaneously our aim was to see if transferable lessons could be learnt regarding how to ensure 'optimal' recruitment to clinical trials.

## Methods

We studied routine consultations between doctors and parents of children with acute lymphoblastic leukaemia (ALL). We collected audio-recordings of these consultations, together with follow-up parent-researcher semi-structured interviews, as part of a wider qualitative study (RAPPORT). This study investigated clinical communication and the parent-practitioner relationship in the care of children with leukaemia with the aim of informing clinical communication practice and training of practitioners. We conducted this study during the period when the UKALL2003 trial (Medical Research Council/National Cancer Research Institute trial) was open to recruitment in the UK. The UKALL2003 trial was a prospective randomised trial investigating if treatment allocation, determined by standardised disease indicators (white cell count and age) plus a molecular test of response to early treatment (minimal residual disease: MRD) could increase survival and minimise toxicity in children with ALL. The trial randomised children at one of two points:

1) If a child was in clinical remission at 28 days of treatment but the MRD showed high levels of residual disease not visible under the microscope, randomisation between the standard treatment (for those in clinical remission) and a more intensive treatment (the intensity of treatment usually given to children who are not in clinical remission at day 28) was offered. This randomisation point occurred approximately four weeks after treatment had begun.

2) If a child was in clinical remission at 28 days, and had low levels of MRD at 28 days and again at 11 weeks, randomisation between standard treatment (according to their clinical disease indicators) and reduced intensity treatment was offered. This randomisation point occurred approximately 12/13 weeks after treatment had begun.

Doctors did not receive payment for recruiting to the trial. Participants were registered in the trial at the time they started treatment, but not all eligible registered participants went on to accept randomisation. Since the decision about entering randomisation is a key one, these decisions were crucial to our investigation.

### Participant recruitment

Participants were recruited to the RAPPORT study from six UK principal treatment centres. Parents were initially approached about RAPPORT by a member of clinical staff and received written information and discussed the study with a RAPPORT researcher. Researchers explained their independence from the clinical teams and that all study information would be kept confidential. Parents who agreed to participate completed a written consent form. Researchers informed parents that they would re-contact them after the audio-recorded consultation to arrange the interview. The study was approved by a UK National Health Service Research Ethics Committee (Ref. 06/MRE08/18).

### Procedure

Sampled consultations included a range of consultations varying from ones that took place immediately before the first set of results that lead to randomisation point 1, to just before the second set of results which lead to randomisation point 2. Parents were interviewed after the audio-recorded consultation. To ensure exploration of core topics, interviewers used a topic guide with prompts about parents' experiences of: communication, consultations including the audiorecorded ones, relationships with staff, and the impact of the illness and treatment on their child (see outline summary in Additional File 1). Trial recruitment was not a pre-planned focus of the overall RAPPORT study or interview questioning. Therefore, discussion of these issues was usually initiated by parents rather than interviewers, although the open-ended and conversational nature of the interviews facilitated parents in introducing or expanding discussion on topics that were important to them. Consultations and interviews were digitally audio-recorded and transcribed. Transcriptions were anonymised. Transcription was verbatim, recording all major disfluencies, emphases and pauses but were stylised in that punctuation was added; for the consultations we also recorded overlapping speech.

### Analysis

The consultation and interview data were linked within cases whereby interview data were considered alongside each corresponding consultation. Analysis was interpretative, but we also considered the subjective meaning of the text to the parents and the doctors. The analysis procedure was informed by the constant comparative approach[15]. The transcripts were analysed for indicators of category relevant to the analytical aims and these categories coded. Categories were both emergent, in that they were grounded in the data, and confirmatory, in that we looked for the presence of factors suggested in the literature as important for optimal recruitment. Identification of category and coding referred to the transcript as a whole, as well as the proximal content of utterances. Codes were compared looking for disparities and consistencies according to: 1) the focus of the consultations particularly whether randomisation was imminent or not, 2) the doctor and 3) the centre. Analyses were conducted by LBD, discussed extensively with KG and BY, and refined in light of these and further discussions with PS and TE. All investigators read several complete transcripts. In presenting our results we use extracts from consultations and interviews to illustrate each core category. These extracts are accompanied with anonymised identification codes indicating centres (A-F) and numeric codes for doctor (D) and parent/family (F). For all extracts, square brackets containing three dots [...] indicate short sections of omitted speech. Square brackets containing text indicate explanation added during transcribing or analysis and capitals were added by transcribers to indicate words emphasised by speakers. While original transcription recorded hesitation less than one second, overlapping speech and disfluency, for ease of reading we removed most of these markers from presented data extracts.

## Results

### Participants

**Figure 1:**
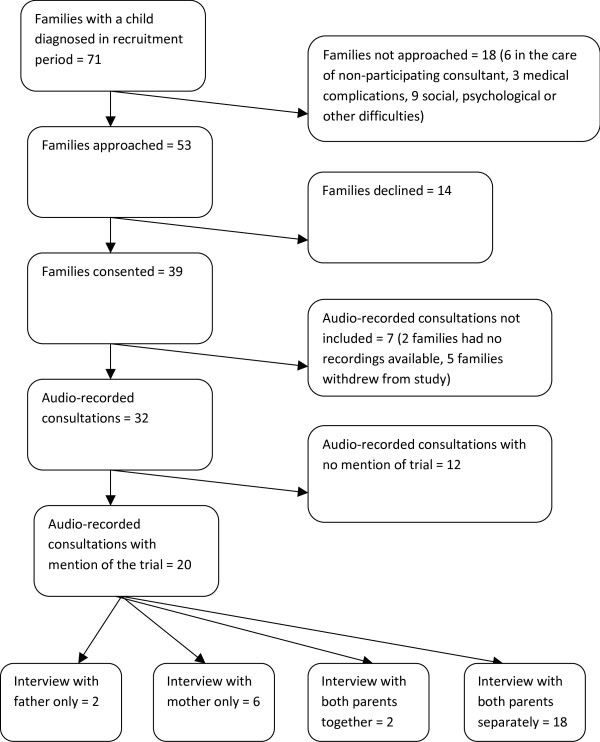
**Participants in consultations and interviews**.

Seventy-one families of newly diagnosed children were cared for during the period when the study was open at the six centres (Figure 1). Of these, 53 families were approached about RAPPORT, 39 consented and 32 had consultations available. Because of the sensitivity of the situation we did not enquire why parents declined participation in RAPPORT, but those who volunteered reasons mainly reported that they were too busy caring for their child. The trial was discussed in 20 consultations which were therefore included in the final analyses for this paper alongside interviews with 30 parents (17 mothers and 13 fathers). The mean lag time from consultation to interview was 17 days (sd = 15 days). Families were from all quintiles of the Townsend Deprivation Index [16] with four from the most and seven from the least deprived quintiles. Children were aged 2 to 11 years, (mean = 5; sd = 3; mode 2 [5 children]). Although participation in RAPPORT was open to anyone, all the families entered in these analysis were white British and had English as their first language. Reflecting the complexity of the topics explored, most interviews lasted over one hour and many lasted more than two.

### How recruitment looked

#### Structure of the consultations

Consultations had different foci: some largely focussed on test results that determined whether a child was eligible for randomisation whilst others were routine clinical visits (Table 1). As Figure 1 illustrates, 62% of all consultations included some discussion about the trial, including randomisation. However, only five included a specific discussion of an *imminent *decision about consent for randomisation. In these five consultations, doctors gave parents the results of the MRD test which ultimately determined whether the child was eligible to be randomised for more intense treatment. In the other 15 consultations doctors and parents discussed future possible randomisation or the rationale for the trial more generally.

**Table 1:** Foci of the consultations

		Audio-recorded consultations (n)
Consultations in which the child was, or could be, eligible for randomisation	High risk MRD - discussion about randomisation to more intensive treatment	5
	
	Low risk MRD - discussion about possible future randomisation to less intense treatment depending on future results	6
	
	Already randomised - discussing new treatment	1
	
	No results yet - discussion about possible future randomisation depending on future results	3

Consultations in which the child was not eligible for randomisation	MRD inconclusive	3
	
	Moved to a more intensive treatment for clinical need	2

A striking feature of consultations was that many were structured in a similar way by doctors and across centres. Doctors led the consultations: they initiated discussions about the trial and the child's clinical management. Unless the consultation focussed on discussion of MRD results, the doctor or parent typically began by summarising the child's progress to date, then the trial was discussed and finally attention returned to the child's progress and well-being. The discussion of the trial was always embedded in the routine clinical 'business' of these consultations. In consultations where MRD results were the main clinical 'business' of the consultation the doctor began by discussing these results. Two crucial issues arise from the structure of the consultations. Firstly, the focus of the consultation, as observed by us, appeared to be the child and not the trial. Secondly, specific discussions around randomisation decisions appeared at a point during the consultation which linked the trial to the child's clinical management. Doctors summarised the treatment to date and then explained how there was uncertainty about how best to proceed and thus introduced why participation in the trial would be an appropriate course of action to consider.

#### Doctors as clinicians and investigators; trials as treatment and research

When discussing the trial, some individual doctors switched between presenting themselves as the manager of the clinical care of the child, who was allied with the parents (clinician role), or as allied with the trial (investigator role). In the clinician role, doctors sometimes emphasised their independence from the trial. For example, a doctor explained to a family how s/he had recommended that another patient leave the trial because "*I think her best interest is, best interests is to be out*." Referring to the trial, this doctor stated "*this is NOT my study" *and emphasised "*my job is to look after her [the patient]" (D/D2)*. Doctors also used language which distanced them from the trial by drawing a contrast (shown in bold) between the perspectives of the clinical team and parents, and the 'perspectives' of the trial:

"But **from our point of view **from looking at it under the microscope **she is in remission**, that means that she's responded, that the leukaemia's responded, most of the cells have gone away. [..] But the wh- point of the- **this leukaemia study **is really **to ask a further question **that **even though the cells that we can see **under the microscope **have gone away **is there still evidence of any leukaemia cells hiding that we can't necessarily see?" (C/D4)

"[using the MRD test] **they're **looking, a much, much better test **than we have **been able to use over the years [...] **they're **looking to see if the leukaemia is above a certain level" (A/D2)

However, sometimes doctors also allied themselves with the trial, using pronouns which implied that they were part of the trial team:

"So what **we're **doing in the UK is **we're **running a trial." (D/D4).

#### Personalisation of treatment and the scientific imperative

Trials such as UKALL2003 aim to create an evidence base for stratifying treatment for children *in the future *but treatments were not being personalised *within the trial*. However, doctors sometimes presented the trial in a way that could be interpreted as a personalisation of treatment. For example,

"And the whole object of this study [trial] is to try and make sure that we give each child the treatment that they need rather than giving every child exactly the same [treatment]" (C/D4).

In contrast, other doctors clearly described the trial as answering a scientific question in the future, rather than providing more personalised treatment for the individual child in the trial:

"I've given you some test results with the understanding of what it means [...] and the way to gain that understanding is by going through the study and so hopefully in five years or so from now we'll KNOW what this means and know whether it's [the MRD test result] important at all and what we should do about it" (B/D1).

#### Uncertainty, clinical equipoise and personal equipoise

For a trial to be deemed ethical there must be uncertainty about whether one treatment is more beneficial than the other. This is termed clinical equipoise. Most doctors readily presented uncertainty about the meaning of the MRD test results saying, for example, *"no [...] we don't know what MRD means"(B/D1)*. They also showed uncertainty about which of the different treatments offered as part of the trial were most effective saying they were *"happy for children in my care to be randomised" *because *"this is a new test" *and *"I'm happy to do either[...] I er believe in both arms of it" (B/D1) *and *"5 years from the beginning [of the trial] look back and say [...] the patients who have more than one in ten thousand [cells remaining after first block of treatment] some of them were on C and some were on B [...] none of them made any difference or C was [better] or B might be better" (D/D2)*.

However, two doctors expressed more certainty about the MRD test. These doctors still presented the equipoise about the *treatment *options but stated that it was known that high risk MRD led to poorer outcomes. One doctor quoted evidence about how remission rates depended on MRD results:

"So 7 out of the 10 children in that group [group in clinical remission with high risk MRD] will never relapse [...][in]the overall group [group in clinical remission without MRD test] we say that about 8 out of 10 children erm with childhood leukaemia will never relapse (F/D2).

Another doctor described how the MRD test could confirm that the child was a low risk patient:

"added on to that test erm is something called minimal residual disease or MRD, which is that test which can test for leukaemia cells erm much better [...] we're going to plan one again for 11 weeks of treatment [...] If it confirms that he is [...] a low risk patient erm we will offer him the possibility of actually getting less treatment than just the standard"(C/D2).

In addition to clinical equipoise some doctors' communication suggested they were in equipoise about treatments *for that individual child *(personal equipoise). For example, a doctor explained that s/he was happy to deliver *"either arm of treatment to [name of child]" *and *"if I wasn't I'd be giving you the one that I thought was better" (D/D1)*.

### How recruitment felt to the parents

Researchers did not specifically ask parents about recruitment to the trial during their follow up interviews so it was they, rather than the researcher, who initiated all discussions about the trial as part of the RAPPORT interview. It also meant that not all of the interviews contained reference to the trial. When parents did discuss the trial their accounts were generally neutral. Parents expressed very little negativity about the trial and the negative comments they did make mostly concerned the trial's procedures such as having to make a very quick decision about randomisation, and being given information about a trial immediately after diagnosis. We found no evidence that the trial *per se *was viewed negatively by the parents, although one did express dissatisfaction with randomisation, which we will discuss later.

#### Parents appeared to understand equipoise, voluntariness and randomisation

Parents appeared to understand that the trial was being conducted because it was uncertain which of two treatments would be most beneficial for patients like their child (clinical equipoise). One father explained how the evidence was not available to support his *"gut reaction" *to *"give him more chemotherapy" *and that the doctor *"said we don't understand the full implications of [the MRD result]" (B/F2)*. A mother put it very succinctly: *"that's the thing with the randomisations they don't have the figures at the moment to say well yes this regime does work better than this regime" (D/F8)*.

However some parents had a preference for higher intensity treatment, despite the information that they had been given about the equipoise of the treatment arms. In explaining why she had consented to randomisation, one mother spoke of how, if her son died and she had not accepted randomisation (and so her son had not a chance of getting a higher intensity of treatment), then she might feel she had not done everything she could to improve his chances of survival:

"If there was a hope that he would get better treatment then at least we felt like we'd done everything that we could [...] if the worst did happen if we'd said regimen A and then he didn't make it I would - I would have always thought what if we'd have done regimen C, would it have made a difference and I think we needed to know that we'd done everything we possibly could" (B/F2).

This mother indicated a preference not to have lower intensity treatment, saying that it would be *"very difficult for me to say, yes, he could just have one [intensive block]" (B/F2)*. A father, who accepted randomisation, commented on his own difficulties in accepting that there was equipoise *"in the back of my mind I can't let go of the thought that two intensive periods is better than one" (E/F1)*.

Parents indicated that they understood that the decision to enter their child in the trial was theirs emphasising how the doctor *"stayed with me for about a good half hour[...] giving me as much information as I wanted before making the decision of whether to randomise or not" (F/F2)*. They also described how doctors told them that they could withdraw their child from the trial: *"of course we were told from day one that we could always pull out of the trial" (C/F5) *and that this would be *"without any repercussion whatsoever" (D/F3)*.

Where parents discussed randomisation they indicated that they understood that randomisation was done by computer: *"It's a bit strange cos it was, like, the computer was making the decision for you you know about your child's treatment and it wasn't like us making the decision or the consultant making a decision." (D/F8) *and that it meant treatment was allocated by chance: *"because we're in a trial, er, as far as I can gather, when you get to a certain point it's like pot luck, you get A or B." (F/F3)*. Although some parents had preferences about treatment arms, as discussed earlier, only one indicated displeasure that randomisation was the method for treatment allocation:

"the computer makes the decision [...] and I just think, they give you all this information and then, you know, randomisation is just purely you're picked at random, it's a lottery. It's er, yeah, I think they should have certain criteria, that maybe if you fit these specific criterias, or if you have a daughter that's fifteen years of age, or twelve years or whatever, a reason for it, or a reason for you not going on it would probably be better rather than just saying 'oh well, the computer picks and that's it.'" (F/F5).

This parent appeared to believe that the treatment was not rationally designed and that his child was receiving a random treatment, rather than one that was rationally designed but allocated at random. His concerns about randomisation may have been linked to this belief.

#### Parents understood the purpose of the trial

Many parents showed that they clearly understood that the trial was trying to balance the potential improvements in survival with the greater risks of serious side-effects from having more intensive treatment:

"we were slightly disappointed that [...] he got regimen A but of course the flip side of the coin is if he gets away with it and he avoids a much more toxic you know regimen then fantastic" (B/F2)

"he [the doctor] was saying, you know "you've got to balance out the low risk here to the high intensity here". Does that balance out? And with her having the [name of serious infection], um, do we really want to put her at risk from being susceptible to those sort of things again when, maybe, she doesn't really - in the bracket that she's in, where it is a 85%, 95% cure rate - do we need to put her in that thing." (F/F5).

Only five of the 30 parents commented on the scientific imperative of the trial, that its aim was to improve treatments in the future, rather than improve treatments for their child. Four of these expressed positive feelings about helping families in other situations, for example:

"at every opportunity that we're able to give something back in terms of information or supporting them in some way we will do it. Um, this is [...] this is such a massive ordeal that anything we can do to make it easier for someone else we will do." (E/F1).

However, one father - from the only family in our sample who discussed the trial in the recorded consultation and declined the randomisation - explained that although he knew the trial *"can help other children in the future" *he felt that *"we've got to look after our child [...] and we didn't want [...] just don't want her to suffer any more" (D/F14)*. This father was clearly indicating that he understood the scientific imperative of the trial and was declining to participate.

#### Parents had some subtle misunderstandings about the trial

The clinical significance of the standard bone marrow tests is well understood but, since the trial was investigating the stratification of treatment on the basis of MRD results, understanding the significance of the MRD test was part of the trial's rationale. However, some parents believed that the MRD test was equivalent to the standard bone marrow test in terms of the certainty about its prognostic meaning and its implications for intensity of treatment. For example, one mother who was told that her child was in clinical remission and was subsequently given low risk MRD results explained how prior to receiving the MRD results she had believed that the MRD *"was obviously going to be good news otherwise he wouldn't be in remission!"(A/F3)*. It seemed she was unclear that the basis of the MRD test is to find evidence of disease that is not visible down the microscope. Analysis of the consultation linked to this interview indicated that, at the start of this consultation, the doctor was aware of the bone marrow results, but not the MRD results. Reflecting this, discussion focussed on the bone marrow result with the doctor emphasising how it indicated that the child had responded well to treatment. About 9 minutes into the consultation the doctor obtained the MRD result. Seeing that it was low risk he explained that the result *"just reinforces that we think he's having a good response to the treatment" (A/D2)*. However, the doctor also went on to explain how the MRD was a way of detecting if there were any leukaemia cells left that could not be seen down the microscope.

Another parent believed that the MRD test, taken early in treatment, indicated a high risk of recurrence:

"When we had the result of the erm the MRD when she had her last bone marrow and it indicated that she was high risk of recurrence, that, that was quite devastating again." (C/F5).

At the time the trial was conducted there were strong indications that the MRD was associated with higher risk of recurrence. Nevertheless, as we explain above, investigating the significance of the MRD test was part of the trial's rationale. The trial aimed to provide experimental evidence of the relationship between the MRD results and clinical recurrence, in the context of the specific treatment regimens. However, this mother seemed to believe the MRD test provided incontrovertible evidence that her child had a *high *risk of recurrence, whereas the MRD, if the relationship was proven, would indicate a *higher *risk of recurrence.

"It was almost like being told, as I said to [name of doctor] that she had leukaemia [...] because it almost we're doing OK and then, you know, we have this high risk of recurrence." (C/F5).

Finally, one mother indicated that because the *"MRD results came back as higher risk, she was randomised to regimen C rather than stay on regimen A"*. She added that she was happy with this treatment allocation because *"the treatment's going to step up a bit hopefully to [...] get the leukaemia under control" (F/F2)*. She believed that her child was receiving more intense treatment as a direct result of the MRD result and, in this sense, that the trial was offering personalisation of treatment for her child rather than creating an evidence base for stratified treatment in the future. She therefore appeared to be confused about the rationale for the randomisation in this part of the trial, which was to investigate whether more therapy would improve outcome for a child with a higher risk MRD result.

In the sampled consultations for these two families, both doctors had expressed certainty about the prognostic value of the MRD test. In the consultation with family C/F5, the doctor stated that it was known that high risk MRD was linked to higher recurrence rates and explained that the trial's purpose was to examine whether altering treatment would improve outcome. In family F/F2's consultation, the doctor also presented certainty, stating that a low risk MRD would increase the future chances of staying disease free and adding that the result *"pushes into, way into, the [...] 90 percents" (F/D3)*. However, neither doctor explicitly stated that the impact of more intense treatment was certain, nor did F/F2's doctor make any statements that implied the trial offered a way of personalising treatment for a child.

## Discussion

In explaining the trial, doctors were subtly, and sometimes implicitly, stressing their dual role and the integration of good clinical care with a clinical trial. Some researchers have called for greater integration of clinical care and research in order to improve accrual [11], but others have voiced concerns about the ethics of doctors recruiting patients for whom they have clinical responsibility [9,17-19]. Because the motivation of the investigator (to answer an empirical question) and the clinician (to manage the clinical care of the child) are different, concerns have revolved around the potential for doctors to unduly influence parental consent when recruiting their own patients [9,19]. It has therefore been suggested that doctors should declare their dual roles [19]. Doctors were not required to overtly declare their dual roles in this trial. However, by using language that sometimes allied themselves with the trial and sometimes with the parents, doctors were imparting information to parents about their dual role, albeit in a subtle way.

The trial discussions were embedded within the consultations and introduced at a time that seemed to link the trial with the child's clinical management. We argue that this served to prioritise the clinical function of the consultation to ensure that the child received the care s/he needs, whilst allowing doctors to introduce the topic of the trial without disrupting this crucial, primary focus. Having the child's clinical management as the focus of the consultation had two implications. First it signified that the trial was answering an important question, which previous research has shown is important in trial recruitment [3]. Second, it meant that there was no disruption of focus; avoiding such disruption has been previously proposed as important in maintaining parent and doctor comfort during trial discussions [3,6].

Of the sampled consultations, 62% had some discussion of the trial. This indicated that doctors were maintaining a regular dialogue about the trial and preparing parents for future decisions, rather than only discussing the trial at decision points. This might normalise being part of a trial and also avoid parents being overwhelmed with new concepts at emotionally difficult times. Our finding that the trial was discussed at so many consultations supports the proposition that studying communication in trials by using just a single, *a priori *identified consultation is likely to overlook these processes [20].

Not all parents initiated discussion of trial participation in their interviews and most of those who did appeared to understand crucial aspects of the trial, particularly randomisation and the separation of clinical care and trial participation. Thus parents either understood the rationale/scientific question and that taking part was their decision or they chose not to mention it. Parents did not comment about the dual role of the doctors. However, a few parents seemed confused about the prognostic significance of the MRD test. In addition, while this particular trial was creating an evidence base about the stratification of treatment in the future, some parents believed that their child's treatment was being personalised as part of the trial. Indeed, some doctors appeared to present the trial in this way. Where parents believed in the prognostic certainty of the MRD test, we have seen that, in the sampled consultations, doctors had presented the MRD test with certainty. In contrast to the confusion about the personalisation of treatment, one father seemed to be concerned that randomisation meant his child was receiving random treatment, rather than random *allocation *of treatment. Parents did not, however, express distress about being invited to participate in a trial or negative views about the overall process of trial recruitment, but some did have concerns about the timing of the approach.

We studied a trial in a specialty with traditionally high accrual rates and which involved delayed randomisation, allowing us to study deliberations about randomisation as well as trial entry *per se*. Crucially, we considered two complementary data streams: observation of the consultations and interviews with the parents. This allowed us to study how doctors presented trial participation, and how the parents understood these discussions and perceived the trial. We looked at trial discussions in a variety of consultations, not just in those in which randomisation decisions were imminent. The variety of consultations was important since there is increasing evidence that understanding the broader context and experience of recruitment is important in understanding the role that communication plays [20]. The way these data were collected is crucial in understanding their significance. The doctors and parents were taking part in a wider study about general communication in the care of children with leukaemia, rather than one specifically focussed on communication about trial recruitment. We believe that this avoided their responses being shaped by how this study was framed and adds to the trustworthiness of our findings. It is particularly noteworthy that parents, in unprompted situation of their interviews, did not harbour generally negative thoughts about being approached to participate, the trial processes or their own decision making.

Studies of recruitment to trials suffer from the likelihood that people who do not want to take part in trials often do not want to take part in other research. This study is no different. Out of 71 families whose child was cared for at the six centres during our recruitment period, most if not all of who were likely to have registered for the UKALL2003 trial, only 32 had consultations recorded. Of these, 12 were ineligible to be included in these analyses because they did not involve a discussion of the trial. Our final sample comprised only white British participants whose first language was English. This may limit the transferability of the findings. Also, this was a late phase trial involving stratification of treatment protocols that were already very successful. Randomisation might not, therefore, be as difficult a decision as in trials where there is a greater uncertainty regarding the outcomes of the different treatment arms [21]. The leukaemia community has seen remarkable improvements in treatments in recent decades, high rates of trial accrual and acceptance of randomisation, a culture of trial involvement among clinical staff and an organisational infrastructure which facilitates trials [22,23]. Our observations might not transfer to trials of treatments in other clinical specialties with different histories, cultural norms and organisation.

### Lessons that could be learnt

This study compared how trial discussions looked to an observer and how they felt to the parents of the children being recruited. Our aim was to identify features of discussions that could promote or inhibit optimal recruitment.

#### Trials can be embedded but caution is needed

The UKALL2003 trial was embedded in clinical practice. It was discussed across many different consultations, introduced at a time that seemed appropriate to clinical care and by the child's consulting physician. A similar, highly integrated, approach has been recently proposed to be a "method for conducting large trials at a low cost" [11]. It might be feared, however, that such conditions would promote high accrual at the expense of voluntariness but we have not found evidence of this. Indeed, the parents showed a generally good understanding of the trial, its separation from routine clinical care and their right to choose to participate or not. The only caveats was that there was some confusion among parents between the creation of an evidence base for the stratification of medicines and the personalisation of treatment for their child and about the experimental nature of the MRD test. Additionally, one father had misunderstood random allocation as random treatment. Our results show that these difficulties might have been linked to the overall complexity of the trial itself and how doctors explained the stratified medicine aspect of the trial. Stratified medicine trials are complex, difficult for doctors to explain, difficult for parents to understand and therefore some confusion is perhaps understandable. Guidelines for doctors on how to explain such trials may be useful. For stratified trials that run for several years, such guidance could be supplemented by updates on important evidence regarding the status of the tests on which the stratification is based. More generally doctors might also consider exploring parents' understanding using sensitive open questions that enable parents to voice their views on key aspects of the trial, before eliciting their decision about randomisation.

#### Doctors can display dual role, equipoise and uncertainty

Doctors in this study displayed two roles: clinician and investigator. At times, they allied themselves with the trial and presented the scientific rationale for the study. At other times they allied themselves with the parents, discussing whether the randomisation was right for the child. We propose that the presentation of these roles created a transparency about the doctors' reasons for involving themselves in research: to reduce treatment uncertainty whilst ensuring that children in their care receive the best treatments. Arguably, doctors' presentation conveyed their goals to parents, which in turn might foster trust. Doctors also showed parents the logic that underpinned their request to enter the child into the trial, i.e., in the absence of evidence about which course of treatment is best it is justifiable to allocate treatment by chance. Perhaps a more explicit method for declaring dual roles at the time of trial recruitment [19] would assist in creating transparency and in fostering trust throughout the process of clinical trial participation.

#### Trial design might assist optimal recruitment

This trial involved delayed randomisation. This might assist parents in distinguishing between clinical care, trials and other forms of medical research. It gives more time for tailored and timely dissemination of trial information, rationale and processes. Previous research has shown that people report the advancement of science as being an important driver in agreeing to take part in trials [24-26]. However, discussing such scientific rationales for trials might disrupt the focus of consultations about the child's treatment and therefore discourage trial participation. The delayed randomisation element of this trial gave doctors opportunities to discuss the scientific imperatives and the science of trials well before they discussed randomisation. Parents therefore had a chance to absorb information about the trial's scientific rationale separately from making the difficult decision about randomisation of their own child.

## Conclusions

A balance of ethical recruitment and high accrual is necessary for optimal recruitment to clinical trials. Studying communication about a trial in the light of two qualitative data streams has enabled us to consider how communication might assist optimal accrual. Doctors presented their dual role subtly and most parents understood randomisation, voluntariness and the scientific imperative of the trial. However, some parents were confused about the purpose of this trial and the experimental status of the MRD test. This might reflect the complexity of this particular trial. Our findings support the embedding of trials in clinical practice but we would urge improved explanation in trials of stratified medicines to avoid confusion about personalisation of treatment.

## Competing interests

LMTBD, PS, KG, TOBE, BY have no competing interests.

## Authors' contributions

TE, BY and PS designed, implemented and monitored the RAPPORT study. BY was principal investigator and KG collected much of the data. LBD led the data analysis and the drafting of the paper. All authors contributed to the data analysis and were involved in writing the paper.

## Authors' information

LMTBD is a chartered psychologist who studies patient perspectives in clinical trials. BY is a chartered psychologist and PS is a clinical psychologist both of whom study communication in health settings. KG is an anthropologist who has studied parent-practitioner relationships in the context of ALL. TOBE is Professor Emeritus of Teenage and Young Adult Cancer and has worked extensively in the clinical treatment of ALL.

## Pre-publication history

The pre-publication history for this paper can be accessed here:

http://www.biomedcentral.com/1471-2288/10/101/prepub

## Supplementary Material

Additional file 1 **Topic Guide - Summary Version**. Outline summary of parent interview topic guide. Click here for file
