# Supplementary material for: Balancing high accrual and ethical recruitment in paediatric oncology: a qualitative study of the 'look and feel' of clinical trial discussions
Source: BMC Med Res Methodol. 2010 Oct 22;10:101. doi: 10.1186/1471-2288-10-101 (PMC2972295; doi:10.1186/1471-2288-10-101)
Supplement: Additional file 1 — Topic Guide - Summary Version. Outline summary of parent interview topic guide. [file 1471-2288-10-101-S1.DOC]

# **Topic Guides – Outline Summary Version**

**Relationships between parents and practitioners regarding**

**children with leukaemia: the Rapport Study**

**Topic Guide for Interviewing Parents**

Interviews with parents included several main topics (indicated in bold) each with a number of sub-topics. The main topics are listed below with examples of sub-topics. These are not exhaustive.

## Demographics and General Background

- I would like to start by getting an overall picture of your family situation?
- I was hoping we could do a little family tree to get an idea of the important people in [child’s name’s] life…
- I would like to ask you a few questions about employment…
- How would you describe your ethnic origin?
- Would you mind if I start by getting an overall picture of the different doctors and nurses that have been involved in [child’s name] care?

## Last Consultation

- Can you tell me a bit about what was on your mind before that last consultation?
- Is there anything that sticks out in your mind about that meeting?
- Do you remember what you were expecting of this consultation?

## Relationship with doctor

- You mentioned earlier some of the other things that were on your mind before you saw Dr [name of lead consultant], were you able to ask about those things?
- How would you say that you and Dr [name of lead consultant] get on together?
- How do you think [child’s name] feels about Dr [name of lead consultant]?
- Looking back to when you first met Dr [name] can you tell me a bit about that first meeting?
- Have your initial impressions of Dr [name of lead consultant] changed at all since then… in what way?
- On the whole how do you usually feel before these consultations?
- Do you think Dr [name of lead consultant] knows you are feeling like that?

## Diagnosis and First Weeks

- Can you tell me something about how you were feeling during those early weeks?
- Was there anyone at the hospital that you could talk to?
- You’ve mentioned that it was Dr [name] who gave the

diagnosis, can you tell me a little more about the time leading up to that? … before you got to the hospital… then when you arrived?

## Parental Concerns about Child

- How would you say all this is affecting [name of child]?

## Part Two - Nurses

## General Background & Last Consultation

- I’d like to ask a few questions about the nurses now if that’s okay.
- What do you think about [name of nurse] being present during consultations?
- Do you ever talk everything through with [name of nurse] after the consultation?
- Are there any things that it’s generally better to ask [name of nurse] about compared to Dr [name of lead consultant]?

## Relationship with nurses

- Looking back to when you first met [name of nurse]… can you tell me a bit about that first meeting?
- Have your initial impressions of [name of nurse] changed at all… in what way?
- Do you think that you and [name of lead nurse] have a reasonable understanding of each other?
- How do you think [child’s name] feels about [name of lead nurse]?

## Nurses on the Ward:

- What kind of things did the nurses do for [child’s name] at the beginning?
- Do you remember any of the nurses in particular from when you were on the ward initially?
- What was it like when you got home and started coming into clinic instead?

## Nurses in Clinic:

- Which of the nurses do you tend to see, when you go to clinic now?
- How do you think [child’s name] feels about [name of clinic nurse X]?
- Are there any questions that it is generally better to ask the nurses in clinic about rather than Dr [name of lead consultant]?

## Community/Macmillan Nurses:

- Do you have a community or Macmillan nurse coming here now?
- Do you feel it’s a help to you that they come out to do the treatment?
- When was last time you had someone round? Do you remember what you talked about or whether you had any specific questions for him/her then?

## Other Staff

- Apart from the doctors and nurses, what are the other members of staff like? Are there any particular members of staff who you see regularly/tend to talk to?

## Advice for Newly Qualified Staff

- If you were advising a recently qualified doctor on the most important things they can do for parents in your situation, what would you say?
- And what would be your advice for a newly qualified nurse?

## Closing Comments

- Is there anything else that you think is important to mention?
- Thank you
- Next steps
